# Bayesian Elastic Net Cox Models for Time-to-Event Prediction: Application to a Breast Cancer Cohort

**DOI:** 10.3390/e28030264

**Published:** 2026-02-27

**Authors:** Ersin Yılmaz, Syed Ejaz Ahmed, Dursun Aydın

**Affiliations:** 1 Department of Statistics, Faculty of Science, Muğla Sıtkı Koçman University, Muğla 48000, Turkey; duaydin@mu.edu.tr; 2 Department of Mathematics and Statistics, Brock University, St. Catharines, ON L2S 3A1, Canada; sahmed5@brocku.ca

**Keywords:** Bayesian elastic net, Cox proportional hazards, high-dimensional survival, shrinkage estimator, posterior contraction

## Abstract

High-dimensional survival analyses require calibrated risk and measurable uncertainty, but standard elastic net Cox models provide only point estimates. We develop a Bayesian elastic net Cox (BEN–Cox) model for high-dimensional proportional hazards regression that places a hierarchical global–local shrinkage prior on coefficients and performs full Bayesian inference via Hamiltonian Monte Carlo. We represent the elastic net penalty as a global–local Gaussian scale mixture with hyperpriors that learn the $\ell_{1}/\ell_{2}$ trade-off, enabling adaptive sparsity that preserves correlated gene groups; using HMC with the Cox partial likelihood, we obtain full posterior distributions for hazard ratios and patient-level survival curves. Methodologically, we formalize a Bayesian analogue of the elastic net grouping effect at the posterior mode and establish posterior contraction under sparsity for the Cox partial likelihood, supporting the stability of the resulting risk scores. On the METABRIC breast cancer cohort (n=1903; p=440 gene-level features after preprocessing, derived from an Illumina HT-12 array with ≈24,000 probes at the raw feature level), BEN–Cox achieves slightly lower prediction error, higher discrimination, and better global calibration than a tuned ridge Cox, lasso Cox, and elastic net Cox baselines on a held-out test set. Posterior summaries provide credible intervals for hazard ratios and identify a compact gene panel that remains biologically plausible. BEN–Cox provides an uncertainty-aware alternative to tuned penalized Cox models with theoretical support, offering modest improvements in calibration and providing an interpretable sparse signature in highly-correlated survival data.

## 1. Introduction

Accurate time-to-event prediction guides decisions on therapy, follow-up, and trial eligibility. Modern molecular profiling yields wide, correlated predictor sets for comparatively modest cohorts, and in this high-dimensional regime, Cox regression can become unstable, and risk scores can miscalibrate. Penalized alternatives such as lasso, ridge, and the elastic net stabilize estimation and encourage structure, yet they return only point estimates and depend on tuning, leaving uncertainty quantification and calibration largely ad hoc. This motivates a Bayesian formulation that delivers calibrated risk and honest uncertainty while respecting correlation among predictors.

The classical model for such time-to-event problems is the Cox proportional-hazards regression [1]. In its original form, the model works well when the number of predictors *p* is small or at most moderate. Modern gene expression studies, however, typically start from tens of thousands of microarray gene-level features measured on only a few thousand patients. In METABRIC, for example, the Illumina HT-12 v3 platform interrogates roughly p≈24,000 probes at the feature level, but the publicly available cBioPortal file that we analyze here already provides gene-level summaries rather than raw feature intensities. After removing genes with missing values in this file, we obtain $p_{\mathrm{raw}}=489$ gene expression variables, and after the 10th-percentile variance filter, we work with p=440 features (see Section 3). Thus, while the original platform is ultra-high-dimensional, our analyzed design matrix has moderate-to-high dimensionality (p=440, n=1903) but remains strongly correlated given *n*, which makes the partial-likelihood surface relatively flat in many directions and the Cox estimates unstable.

Here, one solution could be to add penalties. The lasso shrinks many coefficients exactly to zero, while ridge regression keeps all coefficients but pulls them toward zero. The elastic net (EN) combines the two ideas and tends to keep groups of correlated genes together [2]. In addition, penalized Cox models have been widely used for high-dimensional survival and gene expression data, including lasso and ridge-type penalties [3], as well as comparative studies and cross-validated prognostic pipelines for microarray cohorts [4]. More recently, post-shrinkage strategies have been proposed for high-dimensional Cox models [5]. Related to ridge-type regularization, weighted ridge ideas and post-selection shrinkage strategies based on weighted ridge have also been developed; see Gao et al. [6] and the follow-up line of work on shrinkage and penalty estimation under censoring (see Hossain and Ahmed [7], Ahmed et al. [8]). Still, the elastic net needs cross-validation to pick two tuning numbers and only returns point estimates, so we do not know how much uncertainty we have in each hazard ratio.

A key statistical motivation behind shrinkage is the bias–variance trade-off: in moderate-to-high dimensions, shrinking noisy estimates toward a central value can reduce mean-squared error (see [9] for a modern post-shrinkage view in high-dimensional modelling). This is not only a heuristic idea: the Stein-type shrinkage approach shows that, for multivariate normal means in dimension p≥3, the usual unbiased estimator can be inadmissible and can be improved by shrinkage estimators. The same principle underlies many regression shrinkage methods. In particular, ridge regression can be interpreted as a Bayes estimator under a Gaussian prior, and many Stein-type and empirical Bayes procedures choose the shrinkage intensity by estimating hyperparameters from data. In this sense, modern penalized survival models sit very close to empirical Bayes thinking, even when they are presented as optimization-based methods. The BEN–Cox model introduced here follows this idea directly: the $\ell_{1}/\ell_{2}$ shrinkage strengths are treated as unknown and learned from the data via hyperpriors, providing a principled Bayesian analogue of data-driven shrinkage.

Related Bayesian survival models with grouping and shrinkage priors have been developed for high-dimensional settings, notably by Lee et al. [10], who proposed simultaneous shrinkage and grouping priors for survival prediction with pre-specified group structures. In contrast, our BEN–Cox approach uses an elastic net prior to induce a correlation-driven “grouping effect” (in the elastic net sense of Zou and Hastie [2]) without requiring pre-specified group labels, which is particularly relevant in exploratory omics settings where reliable pathway or module annotations may be incomplete or uncertain.

Bayesian thinking treats a penalty as the negative log of a prior. A Laplace prior gives the Bayesian lasso [11], and mixing a Laplace with a Gaussian prior gives the Bayesian elastic net (BEN) [12,13]. Putting hyperpriors on the mixing scales lets the data decide how much $\ell_{1}$ and $\ell_{2}$ shrinkage to apply, and posterior samples provide full credible intervals. Recent theory also shows that such scale-mixture priors contract toward the truth at near-optimal rates under sparsity [14]. In the survival context specifically, Lee et al. [10] developed Bayesian survival models with simultaneous shrinkage and grouping priors for high-dimensional data, providing an important methodological lineage for shrinkage-based time-to-event modelling. Our BEN–Cox approach builds on this foundation by using elastic net priors that induce correlation-driven grouping without requiring pre-specified group structures.

In this paper, we fit a Bayesian EN-Cox model in the simulation study and to the METABRIC cohort (n=1903, p=440 gene-level features derived from an original ∼24,000-probe array). Our aims are to

- Show that Hamiltonian Monte Carlo can draw stable posterior samples in a correlated, high-dimensional survival setting;
- Compare predictive accuracy, discrimination, and calibration against a tuned ridge Cox, lasso Cox, and elastic net Cox baselines;
- Check that the genes kept by the model contain known biology, for example, the PAM50 signature first reported in Curtis et al. [15]; and
- Provide theoretical support for the BEN–Cox framework by formalizing a Bayesian elastic net grouping effect at the posterior mode and establishing a posterior contraction result under sparsity for the Cox partial-likelihood setting.

Here, it should be noted that this paper’s contribution is methodological development within the existing lineage of Bayesian shrinkage survival models [10,12,13], rather than a fundamentally new framework.

The rest of this article is organized as follows: Section 2 introduces the proposed BEN–Cox model, prior specification, and theoretical properties. Section 3 describes the METABRIC data set and the pre-processing procedure. Section 4 and Section 5 present the application results, including predictive performance metrics and calibration diagnostics based on Demler et al. [16]. Section 6 discusses the implications and limitations of the proposed method, and Section 7 provides concluding remarks.

## 2. Methodology

### 2.1. Model Specification

For patient i∈{1,…,n}, let $\left(y_{i},\delta_{i},x_{i}\right)$ denote the observed data, where $y_{i}$ represents the follow-up time, $\delta_{i}$ is the event indicator ($\delta_{i}=1$ if the event is fully observed and $\delta_{i}=0$ if the event time is right-censored), and $x_{i}$ is a *p*-dimensional vector of *z*-standardized $\log_{2}$-transformed gene expression feature intensities. Here, $\log_{2}$ denotes the base-2 logarithm, corresponding to the conventional $\log_{2}$ transformation applied to raw expression intensity measurements. This transformation is standard in gene expression analysis to stabilize variance across the dynamic range of intensities and to improve the distributional behaviour of the data for downstream modelling [15]. The Cox proportional hazards model [1] assumes

(1) $$h\left(t|x_{i}\right)=h_{0}\left(t\right)\;\exp\;\left(x_{i}^{T}\beta \right),$$

with unspecified baseline hazard $h_{0}\left(t\right)$. Inference uses the log partial likelihood

(2) $$\ell \left(\beta \right)=\sum_{i=1}^{n}\delta_{i}\left\{x_{i}^{T}\beta -\log\;\sum_{j:\;y_{j}\geq y_{i}}\exp\;\left(x_{j}^{T}\beta \right)\right\}.$$

When *p* is large, and predictors are strongly correlated, regularization is essential; more generally, the same framework covers regimes where *p* may grow with *n*.

### 2.2. Bayesian Elastic Net Prior

We place an elastic net prior on β that unifies $\ell_{1}$ and $\ell_{2}$ shrinkage:

(3) $$p\left(\beta |\lambda_{1},\lambda_{2}\right)\;\propto \;\exp\;\left(-\lambda_{1}{∥\beta ∥}_{1}-\frac{\lambda_{2}}{2}{∥\beta ∥}_{2}^{2}\right),$$

recovering lasso and ridge as $\lambda_{2}\to 0$ or $\lambda_{1}\to 0$ [2]. For computation, we use the normal-exponential mixture for the Laplace part with a Gaussian ridge factor:

(4) $$\begin{array}{cc} z_{j}|\lambda_{1} & ∼\mathrm{Exp}\;\left(\frac{\lambda_{1}^{2}}{2}\right),\;j=1,\ldots ,p,\\ \beta_{j}|z_{j},\lambda_{2} & ∼N\;\left(0,\;{\left(\frac{1}{z_{j}}+\lambda_{2}\right)}^{-1}\right), \end{array}$$

which integrates back to (3). We use weakly-informative global hyperpriors,

(5) $$\lambda_{1}∼\mathrm{Half}\;-\;\mathrm{Cauchy}\left(0,5\right),\;\lambda_{2}∼\mathrm{Half}\;-\;\mathrm{Cauchy}\left(0,5\right),$$

so the $\ell_{1}/\ell_{2}$ balance is learned from the data (see Appendix C for a sensitivity analysis regarding the choice of hyperprior scale). Importantly, $\lambda_{1}$ and $\lambda_{2}$ are treated as unknown parameters and inferred jointly with the regression coefficients through this hierarchical hyperprior structure, rather than being manually tuned via cross-validation. The Half-Cauchy(0,5) hyperprior is a standard weakly informative choice for scale parameters [17], though prior sensitivity to this scale choice is possible and warrants consideration in practice. The $\ell_{1}$ part introduces sparsity; the $\ell_{2}$ part stabilizes groups of correlated features (useful for gene modules).

### 2.3. Posterior Distribution

Let $z={\left(z_{1},\ldots ,z_{p}\right)}^{T}$. Up to normalizing constants, the joint posterior is

$$\pi \left(\beta ,z,\lambda_{1},\lambda_{2}|y,\delta ,X\right)\;\propto \;\exp\;\left(\ell (\beta )\right)\;\prod_{j=1}^{p}\phi \;\left(\beta_{j};0,{\left(\frac{1}{z_{j}}+\lambda_{2}\right)}^{-1}\right)\;\frac{\lambda_{1}^{2}}{2}\;e^{-\frac{\lambda_{1}^{2}}{2}z_{j}}\;\pi \left(\lambda_{1}\right)\;\pi \left(\lambda_{2}\right).$$

Because the Cox likelihood is non-conjugate, we use Hamiltonian Monte Carlo (HMC) for full Bayesian inference.

### 2.4. Hamiltonian Monte Carlo Inference

We fit the model with the No-U-Turn Sampler [18,19]. The parameter block is

$$\Theta =(\beta ,\log z,\log\lambda_{1},\log\lambda_{2}),$$

and the target potential U(Θ)=−logπ(Θ∣data) uses (2) and (4). We run four chains, warm up to adapt step size and diagonal metric, then draw posterior samples. Convergence is assessed with $\hat{R}<1.01$ and effective sample size thresholds; energy/BFMI diagnostics are checked for geometry pathologies. We sample the latent scale variables through a log-transformation (i.e., sampling $\log z_{j}$) to enforce positivity and improve numerical stability. Posterior predictive survival uses Breslow’s estimator per draw. In this context, let $y_{\left(1\right)}<\cdots <y_{\left(K\right)}$ denote the distinct ordered event times in the sample, let $d_{k}$ be the number of events at time $y_{\left(k\right)}$, and define the risk set $R\left(k\right)=\left\{j:\;y_{j}\geq y_{\left(k\right)}\right\}$. Then Breslow’s estimator at posterior draw *m* is given by

$${\hat{H}}_{0}^{\left(m\right)}\left(t\right)=\sum_{y_{\left(k\right)}\leq t}\frac{d_{k}}{\sum_{j\in R_{\left(k\right)}}\exp\left(x_{j}^{T}\beta^{\left(m\right)}\right)},\;{\hat{S}}^{\left(m\right)}\left(t|x\right)=\exp\;\left(-{\hat{H}}_{0}^{\left(m\right)}\left(t\right)\;e^{x^{T}\beta^{\left(m\right)}}\right).$$

**Figure 1:**
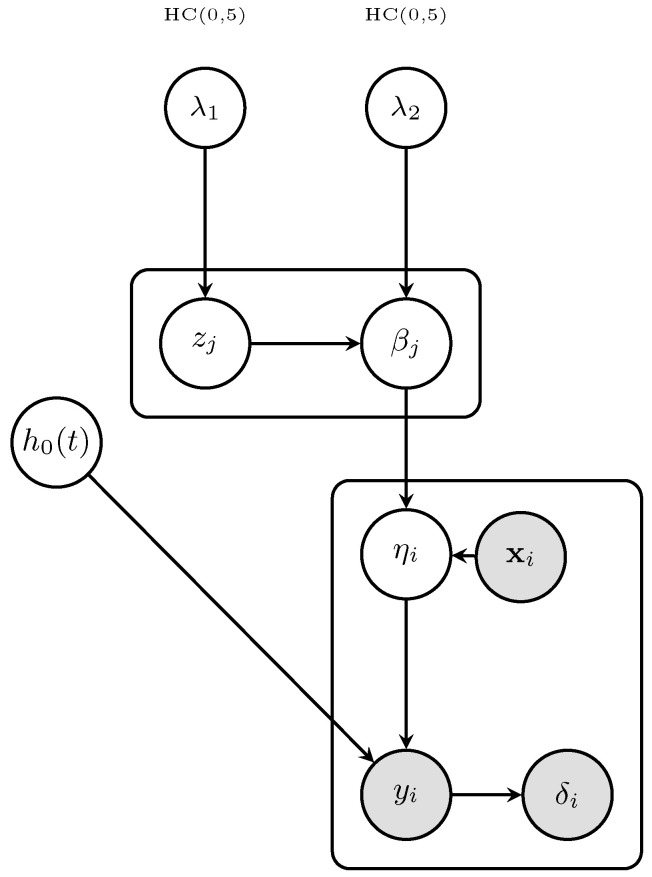
Probabilistic graphical model for the Bayesian elastic net Cox (BEN–Cox) regression framework showing the hierarchical prior structure and survival data likelihood. The model employs a normal-exponential mixture representation of the elastic net prior as specified in Equations (3)–(5). Shaded nodes represent observed variables, while unshaded nodes denote latent variables and parameters. Rectangular plates indicate replication over feature (j) and subject (i) indices.

Before turning to the theoretical properties, we briefly summarize how the proposed procedure works. Figure 1 shows a DAG of the approach, highlighting the parameters used in the model and their probabilistic relationships.

In Figure 1, HC(0,5) is a Half-Cauchy hyperprior with a scale of 5, providing weakly informative priors for the global shrinkage parameters $\lambda_{1}$ and $\lambda_{2}$, as specified in (5). $\lambda_{1}$ is a global $\ell_{1}$ penalty parameter controlling the degree of sparsity in the elastic net prior; learned from the data via the Half-Cauchy(0,5) hyperprior and typically scaling as $\sqrt{(\log p)/n}$ in high-dimensional regimes. $\lambda_{2}$ is again a global $\ell_{2}$ penalty parameter controlling ridge regularization strength; keeping $\lambda_{2}>0$ ensures posterior propriety and stabilizes HMC sampling. $z_{j}$ is a latent exponential scale variable with $z_{j}∼\mathrm{Exp}\left(\lambda_{1}^{2}/2\right)$, enabling the normal-exponential mixture representation of the Laplace component of the elastic net prior in (4). $\beta_{j}$ is the regression coefficient for the *j*-th gene expression feature (covariate $x_{j}$); conditionally Gaussian given $z_{j}$ and $\lambda_{2}$, with $\beta_{j}|z_{j},\lambda_{2}∼N\;\left(0,{\left(1/z_{j}+\lambda_{2}\right)}^{-1}\right)$, as in (4). $h_{0}\left(t\right)$ is an unspecified baseline hazard function in the Cox model (1); estimated post hoc via Breslow’s estimator at each posterior draw. $\eta_{i}$ is our linear predictor for subject *i* and $\eta_{i}=x_{i}^{T}\beta$, representing the log hazard ratio in (1). $x_{i}$: *p*-dimensional vector of *z*-scored $\log_{2}$ gene expression measurements for subject *i*, pre-processed as described in Section 3.4. Finally, $y_{i}$ is an observed survival time for subject *i* possibly right-censored, and $\delta_{i}$ is a known event indicator, with $\delta_{i}=1$ if death is observed and $\delta_{i}=0$ if the observation is right-censored at time $y_{i}$.

### 2.5. Theoretical Properties

This section summarizes the theoretical properties of the introduced BEN–Cox approach. In particular, Theorem 1 gives a Bayesian analogue of the elastic net grouping idea at the posterior mode, and Theorem 2 establishes posterior contraction under sparsity. A complete proof of Theorem 2, including test construction, prior mass, and entropy bounds in the Cox partial-likelihood setting, is provided in Appendix A. In this context, we use the following notation: ${∥\cdot ∥}_{q}$ is the $\ell_{q}$ norm; $I_{p}$ is the p×p identity matrix; $R_{i}=\left\{j:\;y_{j}\geq y_{i}\right\}$ is the Cox risk set. In addition, $I\left(\beta \right)=-\nabla^{2}\ell \left(\beta \right)$ are the observed partial-likelihood information. Posterior is Π(·∣data) under (3) and (4). Also, the following assumptions are needed to ensure the theoretical soundness:

**Assumption** **1.**

*(A1)*  *Columns of X are z-scored on the training split; $∥x_{\cdot j}{∥}_{2}^{2}/n=1$ and entries are bounded.*
*(A2)*  *As a Cox model regularity condition, event times lie in a compact interval, and the baseline hazard is locally bounded; ℓ(β) is twice continuously differentiable (see [1]).*
*(A3)*  *There exists κ>0 such that for all v with support S, $\left|S\right|\leq s_{0}$, $v^{T}I\left(\beta \right)v\geq \kappa {∥v∥}_{2}^{2}$ in a neighbourhood of $\beta^{⋆}$.*
*(A4)*  *As a sparsity assumption, the truth $\beta^{⋆}$ is $s_{0}$-sparse with $s_{0}\log p=o\left(n\right)$.*
*(A5)*  *$\lambda_{1},\lambda_{2}$ follow the half-Cauchy priors in *(5)* with positive density near $\{\lambda_{1}≍\sqrt{(\log p)/n},$ $0<\lambda_{2}\leq C\}$ (see [17]).*

We can explain the details of assumptions individually as follows: (A1) standardizes columns so correlation and curvature arguments are interpretable and avoids scale pathologies in HMC, which is crucial for Bayesian inference. When it comes to (A2), it is the usual smoothness condition that guarantees a well-behaved partial likelihood. (A3) is the high-dimensional analogue of identifiability: restricted curvature prevents flat directions on sparse supports, which can be interpreted as the Cox counterpart of restricted eigenvalues. (A4) encodes the high-dimensional regime with effective dimensionality $s_{0}$; it is the minimal condition for $\sqrt{(s_{0}\log p)/n}$ rates; and finally, (A5) ensures the prior places enough mass near the optimal $\lambda_{1}$ scale while keeping $\lambda_{2}>0$ so the geometry is strongly convex and the posterior proper.

In the following, two basic geometric properties are introduced that we will use repeatedly: (i) the concavity of the Cox partial likelihood, which controls the curvature of the log-posterior, and (ii) the mixture representation of the elastic net prior, which links our hierarchical formulation (4) back to the penalty in (3). These results are standard, but they are provided for completeness.

**Lemma** **1** (Concavity of the Cox partial likelihood)**.** *Under (A2), ℓ(β) in *(2)* is concave in β and $I\left(\beta \right)=-\nabla^{2}\ell \left(\beta \right)$ is positive semi-definite (see Cox [1] for standard log-sum-exp concavity).*

**Lemma** **2.** *The latent parameterization* (4) *integrates to the elastic net prior* (3)*:*

$$\int_{0}^{\infty }\phi \;\left(\beta_{j};0,{\left(\frac{1}{z_{j}}+\lambda_{2}\right)}^{-1}\right)\cdot \frac{\lambda_{1}^{2}}{2}e^{-\left(\lambda_{1}^{2}/2\right)z_{j}}\;dz_{j}\;\propto \;\exp\;\left(-\lambda_{1}\left|\beta_{j}\right|-\frac{\lambda_{2}}{2}\beta_{j}^{2}\right).$$

*See Li and Lin [12] for the normal-exponential representation of Laplace.*

With $\lambda_{2}>0$, the Gaussian ridge factor controls the tails in all directions. Since ℓ(β) is concave (see Lemma 1) and the hyperpriors are proper according to assumption A5, the posterior normalizes. Moreover, $-\ell \left(\beta \right)+\frac{\lambda_{2}}{2}{∥\beta ∥}_{2}^{2}$ is strongly convex with curvature $\geq \lambda_{2}$, so the posterior mode in β is unique. Therefore, this stabilizes the optimization and HMC geometry.

With these building blocks in place, we now formalize two behaviours that are particularly relevant in our high-dimensional gene expression setting. First, the grouping effect shows that highly correlated gene features tend to receive similar coefficients, so the model naturally picks gene modules rather than arbitrary single gene features. Second, a contraction result shows that, under sparsity, the BEN–Cox posterior concentrates around the true coefficient vector at the usual high-dimensional rate, providing reassurance that our calibrated risk scores are statistically well behaved as *n* grows. We state a grouping theorem for the posterior mode, followed by a contraction theorem for the full posterior.

**Theorem** **1** (Bayesian elastic net grouping at the posterior mode)**.** *Assume (A1) and (A2). Let ${\hat{\beta }}_{\mathrm{MAP}}$ be the posterior mode under (3) with fixed $\left(\lambda_{1},\lambda_{2}\right)$ and $\lambda_{2}>0$. For standardized columns j,k with sample correlation $\rho_{jk}$,*

$$\left|{\hat{\beta }}_{\mathrm{MAP},j}-{\hat{\beta }}_{\mathrm{MAP},k}\right|\leq \frac{∥\nabla \ell \left({\hat{\beta }}_{\mathrm{MAP}}\right){∥}_{\infty }+2\lambda_{1}}{\lambda_{2}}\;\sqrt{2(1-\rho_{jk})}.$$

*In particular, if $\rho_{jk}\to 1$ and $(∥\nabla \ell \left({\hat{\beta }}_{\mathrm{MAP}}\right){∥}_{\infty }+2\lambda_{1})/\lambda_{2}$ remains bounded, then ${\hat{\beta }}_{\mathrm{MAP},j}-{\hat{\beta }}_{\mathrm{MAP},k}\to 0$. Hence, the difference tends to zero (Bayesian analogue of the elastic net’s grouping effect; see Zou and Hastie [2], Hans [13]).*

**Proof.**  Consider $Q\left(\beta \right)=-\ell \left(\beta \right)+\lambda_{1}{∥\beta ∥}_{1}+\frac{\lambda_{2}}{2}{∥\beta ∥}_{2}^{2}$. Karush–Kuhn–Tucker (KKT) conditions give $0=-\nabla \ell \left({\hat{\beta }}_{MAP}\right)+\lambda_{1}s+\lambda_{2}{\hat{\beta }}_{MAP}$ with $s_{j}\in \left[-1,1\right]$. Taking the *j*–*k* difference,

$$\lambda_{2}\left({\hat{\beta }}_{MAP,j}-{\hat{\beta }}_{MAP,k}\right)={\left[\nabla \ell \left({\hat{\beta }}_{MAP}\right)\right]}_{j}-{\left[\nabla \ell \left({\hat{\beta }}_{MAP}\right)\right]}_{k}-\lambda_{1}\left(s_{j}-s_{k}\right),$$

so $|{\hat{\beta }}_{MAP,j}-{\hat{\beta }}_{MAP,k}|\leq \frac{{|\left[\nabla \ell \right]}_{j}-{\left[\nabla \ell \right]}_{k}|}{\lambda_{2}}+\frac{2\lambda_{1}}{\lambda_{2}}.$ For the score difference, express the Cox score using risk-set weights and apply Cauchy–Schwarz to the difference column $d=x_{\cdot j}-x_{\cdot k}$. With standardization (A1), ${∥d∥}_{2}^{2}=2n\left(1-\rho_{jk}\right)$, yielding ${|\left[\nabla \ell \right]}_{j}-{\left[\nabla \ell \right]}_{k}|\leq \left(\sum_{i}\delta_{i}\right)\sqrt{2n(1-\rho_{jk})}.$ Scaling per observation and absorbing $\sum_{i}\delta_{i}/n=O\left(1\right)$ gives the stated bound with $∥\nabla \ell \left({\hat{\beta }}_{MAP}\right){∥}_{\infty }/\lambda_{2}\cdot \sqrt{2(1-\rho_{jk})}$. If $\rho_{jk}\to 1$ and $\lambda_{1}/\lambda_{2}$ is not diverging, the difference vanishes. Since the posterior typically concentrates in a neighbourhood of ${\hat{\beta }}_{\mathrm{MAP}}$ under standard regularity, the grouping behaviour at the mode is often reflected in posterior summaries as well; we observe this empirically in the METABRIC analysis.    □

Here we should emphasize that Theorem 1 is a statement at the posterior mode. While the posterior distribution is (locally) log-concave and concentrates around ${\hat{\beta }}_{MAP}$ under standard regularity conditions, the extension to posterior means is supported only by heuristic arguments: highly correlated gene features tend to have similar posterior mean coefficients in practice, as we observe empirically in the METABRIC analysis. No formal theoretical result is provided for posterior means; see Li and Lin [12], Hans [13] for related discussion of Bayesian elastic net concentration.

**Theorem** **2** (Posterior contraction)**.** *Assume (A1)–(A5) and let $\epsilon_{n}≍\sqrt{(s_{0}\log p)/n}$, where *≍* denotes that both sides are bounded by constant multiples of each other (i.e., the same order of magnitude). Then there exists M>0 such that*

$$\Pi \;\left(∥\beta -\beta^{⋆}{∥}_{2}<M\epsilon_{n}\;|\;y,\delta ,X\right)\;\overset{P}{\to }\;1.$$

This theorem is a posterior contraction result: as *n* grows, the BEN–Cox posterior for β concentrates inside an $\ell_{2}$-ball of radius $\epsilon_{n}≍\sqrt{(s_{0}\log p)/n}$ around the sparse truth $\beta^{⋆}$. The proof follows the standard Bayesian contraction arguments of Ghosal and van der Vaart [14], adapted to the Cox partial-likelihood setting, and combines three ingredients. (i) Tests: Assumption (A3) provides local quadratic lower bounds for ℓ(β), which yield exponential tests that separate $\beta^{⋆}$ from the complement of an $\ell_{2}$-ball of radius $M\epsilon_{n}$. (ii) Prior mass: By Lemma 2 and (A5), the prior allocates at least $\exp(-Cs_{0}\log p)$ mass to such balls (taking $\lambda_{1}≍\sqrt{(\log p)/n}$ and using $\lambda_{2}>0$ to stabilize curvature). (iii) Complexity: The class of $s_{0}$-sparse vectors within radius $M\epsilon_{n}$ has logarithmic complexity of order $s_{0}\log p$, which is dominated by $n\epsilon_{n}^{2}$. A complete proof specialized to our Cox partial-likelihood setting (including the testing argument and the hyperprior treatment) is given in Appendix A.

Under Assumptions (A1)–(A4) and the contraction result in Theorem 2, the posterior for $\left(\beta ,H_{0}\right)$ concentrates around the true pair $\left(\beta^{⋆},H_{0}^{⋆}\right)$. The survival function $S(t|x;\beta ,H_{0})$ is continuous in $\left(\beta ,H_{0}\right)$ on compact time intervals, and Breslow’s estimator is uniformly consistent for the baseline cumulative hazard in the Cox model [20]. Combining these facts, one can obtain:

$$\underset{t\in [0,\tau ]}{\sup}\left|E\left[{\hat{S}}^{\left(m\right)}\left(t|x\right)|\mathrm{data}\right]-S_{0}\left(t|x\right)\right|\overset{P}{\to }0,$$

for any fixed covariate vector x and finite horizon τ. In other words, the posterior predictive survival curves converge to the true survival function uniformly on compact time intervals.

From a computational point of view, the ridge component with $\lambda_{2}>0$ induces strong convexity in β, while the *z*-scaling in (A1) keeps the design entries bounded [18,19].

For practical implementation, it is helpful to summarize the full BEN–Cox workflow from raw gene expression data to posterior summaries and predictive metrics. The following step-by-step Algorithm 1 makes explicit how the model, prior, MCMC inference, and evaluation pieces fit together in our analysis.

**Algorithm 1** BEN–Cox pipeline  **Input:** gene matrix X, times y, events δ.  **Output:** posterior draws ${\left\{\beta^{\left(m\right)}\right\}}_{m=1}^{M}$, test-set performance metrics.  1. Pre-processing      1.1 Remove gene-level features whose variance is below the 10th percentile [15].      1.2 *z*-score each column of X using training-set moments.  2. Train–test split      2.1 Randomly split subjects 80/20 into training and test sets, stratified by δ.      2.2 Fix the random seed and record the split for reproducibility.  3. Model and prior      3.1 Specify the Cox model (1) with elastic net prior (3).      3.2 Use the normal-exponential mixture representation (4) and          Half-Cauchy hyperpriors for $\left(\lambda_{1},\lambda_{2}\right)$ as in (5).  4. HMC sampling with Stan (training set only)      4.1 Fit the model using 4 chains, warm-up + sampling.      4.2 Check convergence: $\hat{R}<1.01$ and ESS >400 for all coefficients.      4.3 Retain posterior draws $\left\{\beta^{\left(m\right)}\right\}$ and, for each draw, compute          the Breslow baseline cumulative hazard ${\hat{H}}_{0}^{\left(m\right)}\left(t\right)$.  5. Prediction on the test set      5.1 For each test subject *i* and draw *m*, compute          linear predictor $\eta_{i}^{\left(m\right)}=x_{i}^{T}\beta^{\left(m\right)}$ and          survival curve ${\hat{S}}^{\left(m\right)}\left(t|x_{i}\right)=\exp\left(-{\hat{H}}_{0}^{\left(m\right)}\left(t\right)e^{\eta_{i}^{\left(m\right)}}\right)$.      5.2 Form posterior summaries (e.g., mean or median survival probabilities).  6. Performance metrics (test set)      6.1 Compute the integrated Brier score (IBS).      6.2 Compute the concordance index (C-index) for discrimination.      6.3 Compute calibration slope and intercept, following Demler et al. [16].

## 3. Data

### 3.1. Cohort Description

We analyze the Molecular Taxonomy of Breast Cancer International Consortium (METABRIC) cohort [15,21]. The study combines tumours from two centres (Cambridge, UK and Vancouver, Canada) and is publicly available through the cBioPortal interface [22]. The dataset version used here is the combined METABRIC cohort as available on cBioPortal (Breast Invasive Carcinoma, METABRIC, Nature 2012 & Nat Commun 2016), which includes both the original discovery cohort and subsequent updates. The dataset contains clinical variables, overall survival, and a gene expression matrix. From this file, we extract overall survival and gene expression information and remove subjects with missing survival time or status. After this quality control step, we retain n=1903 primary invasive breast cancers for our analysis.

### 3.2. Outcome

The time-to-event endpoint is overall survival (OS). In the cBioPortal file, OS is reported in months; we convert this to days for the modelling step and define the event indicator $\delta_{i}=1$ for deaths and $\delta_{i}=0$ for right-censored observations. Among the 1903 subjects, 800 experience the event (42.0%), and the median follow-up is approximately 16.4 years, estimated using the reverse Kaplan–Meier method.

### 3.3. Predictors

- Gene expression: The METABRIC dataset provides Illumina HT-12 v3 microarray measurements summarized at the gene level. In the version used here, this block contains $p_{\mathrm{raw}}=489$ gene expression features, indexed from the BRCA1 gene onward. While the original METABRIC platform measures many more microarray gene-level features, we restrict attention to this gene-level expression matrix for a more stable and reproducible design.

### 3.4. Preprocessing

We first remove genes whose variance lies below the 10th percentile across patients, leaving p=440 gene expression features. This variance filter removes almost-constant gene features and improves the conditioning of the design matrix. The data are then split once into an 80% training set and a 20% test set, stratified by the event indicator so that the censoring fraction is similar in both splits. Each gene is *z*-scored in the training set to have mean 0 and variance 1, and the same centring and scaling are applied to the test set. Finally, the survival tuples $\left(y_{i},\delta_{i}\right)$ are encoded as described in Section 2.1, with $y_{i}$ measured in days and $\delta_{i}$ the event indicator.

## 4. Simulation Study

To evaluate the finite-sample performance of the proposed BEN–Cox model under controlled conditions, we conduct a simulation study that varies the dimensionality ratio p/n and correlation structures among predictors. We compare BEN–Cox against three baseline methods: ridge Cox, lasso Cox, and frequentist elastic net Cox (EN-Cox), all implemented via glmnet [3] with penalty parameters selected by five-fold cross-validation.

### 4.1. Simulation Design

We generate survival data from a Cox proportional hazards model with true hazard function $h\left(t|x_{i}\right)=h_{0}\left(t\right)\exp\left(x_{i}^{T}\beta^{⋆}\right)$, where $h_{0}\left(t\right)=1$ (constant baseline hazard). The true coefficient vector $\beta^{⋆}$ is $s_{0}$-sparse, with $s_{0}=10$ non-zero entries set to $\beta_{j}^{⋆}=0.5$ for j=1,…,10 and $\beta_{j}^{⋆}=0$ otherwise.

We consider the following simulation scenarios:

- Dimensionality: (n,p)∈{(150,50),(150,150),(300,50),(300,150)}, corresponding to p/n ratios ranging from 1/3 to 1 to align the real data example and simulation settings.
- Correlation structures:
  1. Independent: $\mathrm{Cov}\left(X_{j},X_{k}\right)=1_{j=k}$
  2. Toeplitz (AR-1): $\mathrm{Cov}\left(X_{j},X_{k}\right)=\rho^{\left|j-k\right|}$ with ρ=0.5
  3. Block-correlated: Predictors grouped into blocks of size 10 with within-block correlation ρ=0.8

For each scenario, we generate B=50 replicate datasets. Covariates $x_{i}$ are drawn from N(0,Σ) with the specified correlation structure Σ, and then *z*-standardized. Event times are generated as $T_{i}∼\mathrm{Exp}\left(\exp\left(x_{i}^{T}\beta^{⋆}\right)\right)$. Censoring times were generated independently from $Uni(0,\tau_{c})$, where $\tau_{c}$ was chosen to give approximately 40% censored observations, comparable to the METABRIC cohort. The observed data are $\left(Y_{i},\delta_{i}\right)=\left(\min\left(T_{i},C_{i}\right),1\left\{T_{i}\leq C_{i}\right\}\right)$.

### 4.2. Evaluation Metrics

We evaluate each method using the following metrics, computed on a held-out test set generated from the same distribution:

- **Estimation error:** $∥\hat{\beta }-\beta^{⋆}{∥}_{2}$ (for BEN–Cox, we use the posterior mean).
- **Variable selection:** Sensitivity (true positive rate) and specificity (true negative rate) for identifying non-zero coefficients. For BEN–Cox, a coefficient is deemed selected if its 95% credible interval excludes zero.
- **Prediction:** Integrated Brier Score (IBS) and C-index on the test set.
- **Sparsity:** Number of selected variables ($N_{\mathrm{selected}}$), compared to the true sparsity $s_{0}=10$.

### 4.3. Simulation Results

Table 1 summarizes the simulation results for the scenario with (n,p)=(300,150) and Toeplitz correlation (ρ=0.5).

**Figure 2:**
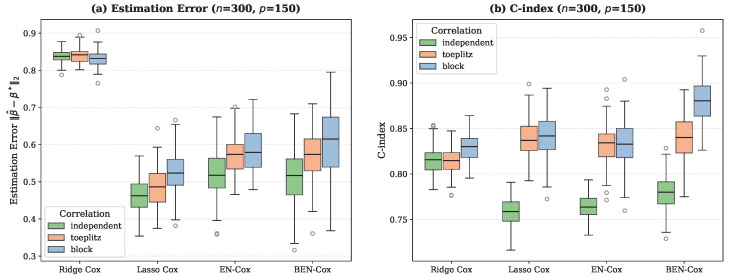
Simulation results for (n,p)=(300,150): (**a**) Estimation error $∥\hat{\beta }-\beta^{⋆}{∥}_{2}$ and (**b**) C-index across 50 replicates for different correlation structures. BEN–Cox achieves the highest C-index across all correlation structures while maintaining competitive estimation error.

In terms of predictive performance, BEN–Cox achieves the highest C-index across all scenarios. In the Toeplitz scenario, BEN–Cox attains the C-index of 0.844±0.002 compared to 0.830±0.002 for lasso Cox and 0.828±0.002 for EN-Cox, representing an improvement of approximately 1.5–2%. This advantage is consistent across correlation structures (Figure 2b), with BEN–Cox showing particularly strong performance under block correlation where the grouping effect (Theorem 1) is most relevant.

Regarding the variable selection, BEN–Cox achieves substantially better specificity than both lasso Cox and EN-Cox across all correlation structures. In the Toeplitz scenario, BEN–Cox attains a specificity of 0.98±0.00 compared to 0.86±0.01 for lasso Cox and 0.81±0.01 for EN-Cox. This translates to BEN–Cox selecting on average 10.9 variables (very close to the true $s_{0}=10$), while lasso Cox selects 22.8 and EN-Cox selects 26.5. Importantly, BEN–Cox also achieves the highest sensitivity (0.99±0.01), demonstrating that the improved specificity does not come at the cost of missing true signals.

Table 2 and Table 3 present results under block correlation and independent predictors, respectively. Under block correlation (ρ=0.8), all methods achieve higher C-index values due to the stronger grouped signal structure; notably, BEN–Cox shows its largest advantage in this setting (C-index 0.883 vs. 0.868 for lasso), which aligns with the grouping effect formalized in Theorem 1. Under independent predictors, C-index values are lower across all methods as there is no correlation structure to exploit, yet BEN–Cox still achieves the best discrimination (0.778 vs. 0.763 for lasso). Importantly, BEN–Cox maintains near-perfect specificity (≥0.98) and near-optimal sparsity ($N_{\mathrm{sel}}\approx 10.8$–11.0, close to the true $s_{0}=10$) across all three correlation structures, demonstrating robustness of its variable selection properties regardless of the underlying predictor correlation.

**Figure 3:**
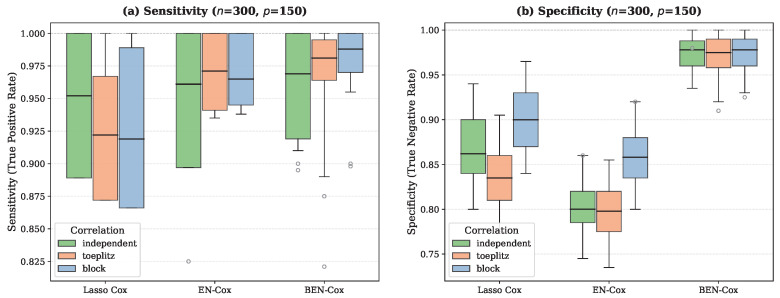
Variable selection performance for (n,p)=(300,150): (**a**) Sensitivity and (**b**) Specificity across 50 replicates. BEN–Cox achieves near-perfect specificity (>0.97) across all correlation structures while maintaining the highest sensitivity. In contrast, lasso Cox and EN-Cox show lower specificity (0.78–0.88), leading to substantially more false positives.

Figure 2 shows boxplots of estimation error and C-index across simulation replicates for different correlation structures at (n,p)=(300,150). Figure 3 compares sensitivity and specificity for the three methods that perform variable selection (lasso Cox, EN-Cox, and BEN–Cox). 

**Figure 4:**
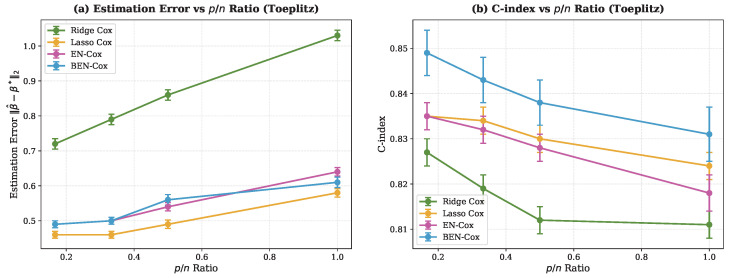
Performance as a function of p/n ratio under Toeplitz correlation: (**a**) Estimation error and (**b**) C-index. BEN–Cox consistently achieves the highest C-index across all p/n ratios examined, with the advantage being most pronounced at higher dimensionality ratios.

Also, BEN–Cox achieves competitive estimation error (0.557±0.011), comparable to lasso Cox (0.495±0.010) and EN-Cox (0.535±0.009), and substantially better than ridge Cox (0.857±0.008). The slightly higher estimation error compared to the lasso Cox reflects the more aggressive shrinkage of noise coefficients toward zero, which contributes to the superior variable selection. When it comes to scalability with p/n ratio, Figure 4 shows that BEN–Cox maintains its performance advantages as the p/n ratio increases from 1/3 to 1. The C-index advantage of BEN–Cox is consistent across all dimensionality regimes, demonstrating robustness to increasing dimensionality.

Overall, the simulation study demonstrates that BEN–Cox provides favourable performance for high-dimensional survival analysis: it achieves the best predictive discrimination (highest C-index), near-optimal variable selection (specificity >0.97, selected model size $\approx s_{0}$), and competitive estimation error. The Bayesian formulation additionally provides full posterior uncertainty quantification for hazard ratios and survival curves, which is not available from the frequentist alternatives.

## 5. Real Data Example: METABRIC Data Analysis

### 5.1. Experimental Design

We treat METABRIC as a single large cohort and mimic a typical prognostic modelling workflow: a model is fitted once on a development set and then evaluated on a separate test set. The data are therefore split once into 80% training and 20% testing, stratified on the event indicator so that the proportion of deaths and censored observations remains comparable across the two splits.

All tuning and model fitting are performed using only the training data. For the frequentist penalized Cox baselines (ridge, lasso, elastic net), we use five-fold cross-validation to select penalty parameters, with the concordance index as the optimization criterion. In contrast, the Bayesian elastic net Cox (BEN–Cox) model treats the elastic net penalties as random: the global shrinkage parameters $\left(\lambda_{1},\lambda_{2}\right)$ follow Half-Cauchy hyperpriors and are learned jointly with the regression coefficients. This avoids an additional tuning loop and lets the data determine the balance between $\ell_{1}$ and $\ell_{2}$ shrinkage.

Posterior inference for BEN–Cox relies on Hamiltonian Monte Carlo as implemented in Stan, using the No-U-Turn Sampler (NUTS) with adaptively tuned step size and diagonal mass matrix. Starting from zero coefficients and diffuse initial values for $\left(\lambda_{1},\lambda_{2}\right)$, the chain is run until trace plots stabilize and effective sample sizes for the regression coefficients are comfortably above standard thresholds. The initial part of the chain is discarded as warm-up, and all posterior summaries are based on the retained draws. In particular, we monitor mixing and autocorrelation for representative coefficients and for the global shrinkage parameters to ensure that the posterior exploration is adequate for downstream prediction and uncertainty quantification.

### 5.2. Baseline Models

To evaluate the BEN–Cox model’s performance, we compare it against four baseline Cox models:

- **Null Cox**: a Cox model with no gene expression covariates, i.e., all subjects share the same baseline hazard $h_{0}\left(t\right)$ (no covariate effects). This provides a no-information baseline for discrimination and absolute-risk calibration.
- **Ridge Cox**: an $\ell_{2}$-penalized Cox model fitted with glmnet, where the penalty $\lambda_{\mathrm{ridge}}$ is chosen by five-fold cross-validation on the training set.
- **Lasso Cox**: an $\ell_{1}$-penalized Cox model fitted with glmnet, with penalty chosen by five-fold cross-validation. This provides a sparse frequentist baseline.
- **EN-Cox (freq)**: a frequentist elastic net Cox model fitted with glmnet, with α=0.5 and penalty chosen by cross-validation. This serves as the direct frequentist counterpart to BEN–Cox.

Also note that all models are fitted on exactly the same training data and are evaluated on the same held-out test set.

### 5.3. Evaluation Metrics

We assess predictive performance in three ways: time-dependent prediction error, discrimination between subjects with different risk, and calibration of predicted survival probabilities.

Let $T_{i}$ denote the true event time for patient *i*, $C_{i}$ the censoring time, and $Y_{i}=\min\left(T_{i},C_{i}\right)$ the observed time, with $\delta_{i}=1\left\{T_{i}\leq C_{i}\right\}$ the event indicator. For a given model, let ${\hat{S}}_{i}\left(t\right)$ be the predicted survival probability at time *t* for patient *i*, and let $\eta_{i}$ be its linear predictor or risk score.

As noted in Algorithm 1, we have three main performance metrics: IBS, C-index, and calibration slope. These metrics are defined as follows:

Integrated Brier score:

The Brier score at time *t* is a squared prediction error adapted to the survival setting. Following the inverse-probability-of-censoring weighting (IPCW) approach, we write

$$\mathrm{BS}\left(t\right)=\frac{1}{n}\sum_{i=1}^{n}\left[\frac{1\{Y_{i}\leq t,\;\delta_{i}=1\}}{\hat{G}\left(Y_{i}-\right)}{\left(0-{\hat{S}}_{i}\left(t\right)\right)}^{2}+\frac{1\{Y_{i}>t\}}{\hat{G}\left(t\right)}{\left(1-{\hat{S}}_{i}\left(t\right)\right)}^{2}\right],$$

where $\hat{G}$ is an estimate of the censoring survival function (e.g., Kaplan–Meier for $C_{i}$). Intuitively, patients who are still under follow-up at time *t* contribute squared error for predicting survival, and patients who die before *t* contribute squared error for predicting death, with both contributions reweighted to account for censoring.

To summarize performance over a time window [0,τ] we use the integrated Brier score (IBS),

$$\mathrm{IBS}\left(\tau \right)=\frac{1}{\tau }\int_{0}^{\tau }\mathrm{BS}\left(t\right)\;dt,$$

which can be interpreted as a time-averaged mean-squared prediction error for survival probabilities up to horizon τ. In practice, we approximate the integral numerically on a fine grid of time points.

Concordance (C) index:

Discrimination is quantified using Harrell’s concordance index (C-index). The C-index estimates the probability that, for a randomly chosen pair of comparable patients (i,j), the one who dies first also has the higher predicted risk. Formally,

$$\hat{C}=\frac{\sum_{i<j}1\left\{Y_{i}<Y_{j},\;\delta_{i}=1\right\}\;1\left\{\eta_{i}>\eta_{j}\right\}+1\left\{Y_{j}<Y_{i},\;\delta_{j}=1\right\}\;1\left\{\eta_{j}>\eta_{i}\right\}}{\sum_{i<j}1\left\{Y_{i}<Y_{j},\;\delta_{i}=1\right\}+1\left\{Y_{j}<Y_{i},\;\delta_{j}=1\right\}},$$

where the denominator counts all comparable pairs (those with an observed event before the other’s follow-up has finished) and the numerator counts how often the model orders their risk correctly.

Grouped calibration (GND $\chi^{2}$):

In addition to the calibration slope and intercept, we also report a Greenwood–Nam–D’Agostino (GND) type grouped-calibration chi-square type statistic [16] as a global calibration measure. To achieve that, we consider τ, which means a fixed, clinically relevant follow-up time (in this study, horizon τ=5 years), and we only look at whether each patient has experienced the event before this time or not.

For each patient, we define a horizon event indicator.

$$Z_{i}\left(\tau \right)=1\left\{T_{i}\leq \tau ,\;\delta_{i}=1\right\},$$

which is 1 if the event occurs on or before time τ and 0 otherwise. We then group patients into *K* risk groups (for example, deciles, K=10) according to their predicted event probabilities ${\hat{\pi }}_{i}\left(\tau \right)$. Let $G_{k}$ denote the index set of patients in group *k*, $n_{k}=\left|G_{k}\right|$ be the group size, and

$$o_{k}=\sum_{i\in G_{k}}Z_{i}\left(\tau \right),\;\bar{Z}=\frac{1}{n}\sum_{i=1}^{n}Z_{i}\left(\tau \right),\;e_{k}=n_{k}\;\bar{Z}$$

be the observed and expected numbers of events in group *k*. The GND statistic is then given by

$$\mathrm{GND}=\sum_{k=1}^{K}\frac{{\left(o_{k}-e_{k}\right)}^{2}}{e_{k}},$$

which summarizes observed and expected event counts across the risk groups; smaller GND values indicate better global calibration of absolute risk at horizon τ. Please note that this is a simplified Greenwood–Nam–D’Agostino-type statistic, using the overall event rate as the reference expected rate.

Expected Calibration Error (ECE):

Following Guo et al. [23], we also report the expected calibration error (ECE), which is a weighted average of the absolute difference between predicted probabilities and observed frequencies across bins:

$$\mathrm{ECE}=\sum_{k=1}^{K}\frac{n_{k}}{n}\left|{\bar{\pi }}_{k}-{\bar{o}}_{k}\right|,$$

where ${\bar{\pi }}_{k}$ is the mean predicted probability in bin *k* and ${\bar{o}}_{k}$ is the observed event frequency. Lower ECE indicates better calibration.

Calibration:

Calibration asks whether predicted risks match observed event frequencies. We focus on a clinically relevant time horizon τ (e.g., 5 years) and consider the predicted event probability ${\hat{\pi }}_{i}\left(\tau \right)=1-{\hat{S}}_{i}\left(\tau \right)$ for each patient. Following Demler et al. [16], we fit a recalibration model of the form

$$\mathrm{logit}\left\{\mathrm{Pr}\left(T_{i}\leq \tau |{\hat{\pi }}_{i}\left(\tau \right)\right)\right\}=\alpha_{0}+\alpha_{1}\mathrm{logit}\left({\hat{\pi }}_{i}\left(\tau \right)\right),$$

with suitable weighting to account for censoring at τ. A calibration slope $\alpha_{1}$ close to one and an intercept $\alpha_{0}$ close to zero indicate that the model’s predicted risks agree well with the observed data; values $\alpha_{1}<1$, for example, suggest that the model overstates differences between low- and high-risk patients.

Bootstrap uncertainty:

To quantify uncertainty in these performance estimates, we use paired bootstrap resampling of the test set. In each of *B* bootstrap replicates, we resample test patients with replacement, recompute the IBS, C-index, and calibration parameters $\left(\alpha_{0},\alpha_{1}\right)$ for each model, and then summarize the resulting distribution by its mean and standard error. This provides a simple way to assess the sampling variability of the performance metrics and to compare BEN–Cox with the frequentist baselines.

### 5.4. Results

This section reports the METABRIC data analysis results for the Bayesian elastic net Cox (BEN–Cox) model and the four baseline Cox models on the held-out test set. A brief summary of the cleaned dataset is given in Table 4 to keep the analysis context clear.

**Figure 5:**
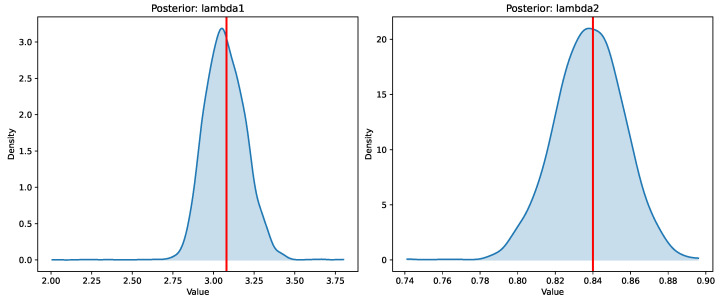
Posterior distributions of the global shrinkage parameters $\lambda_{1}$ (L1/sparsity) and $\lambda_{2}$ (L2/ridge). The posteriors show that the data favour a combination of both penalty types, with the L1 component providing sparsity and the L2 component handling correlated predictors. Red line indicates the selected λ values.

The final BEN–Cox model is fitted with Stan using Hamiltonian Monte Carlo (HMC). Figure 5 shows the posterior distributions of the global shrinkage parameters, which concentrate around $\lambda_{1}\approx 3.08$ (L1 component) and $\lambda_{2}\approx 0.84$ (L2 component), indicating a non-trivial combination of sparsity-inducing and ridge-type shrinkage.

Convergence diagnostics for the fitted model are provided in Table 5. The split-$\hat{R}$ values are essentially 1.00 for the median regression coefficient and for both global penalty parameters, and the effective sample sizes (ESSs) are in the low thousands, which indicates stable posterior exploration. Therefore, it can be said that the model parameters have converged well. Finally, although we estimate the recalibration intercept and slope $\left(\alpha_{0},\alpha_{1}\right)$ for each model using the scheme in Section 5.3, we do not tabulate them here; instead, we summarize calibration through the GND $\chi^{2}$ statistic and ECE in Table 6.

Please note that as an implementation detail, for sampling we use 4 chains with 2000 iterations each (1000 warm-up + 1000 sampling). The total pipeline runtime was approximately 3 hours on standard hardware (Intel i7, 24 GB RAM) using only the CPU.

**Figure 6:**
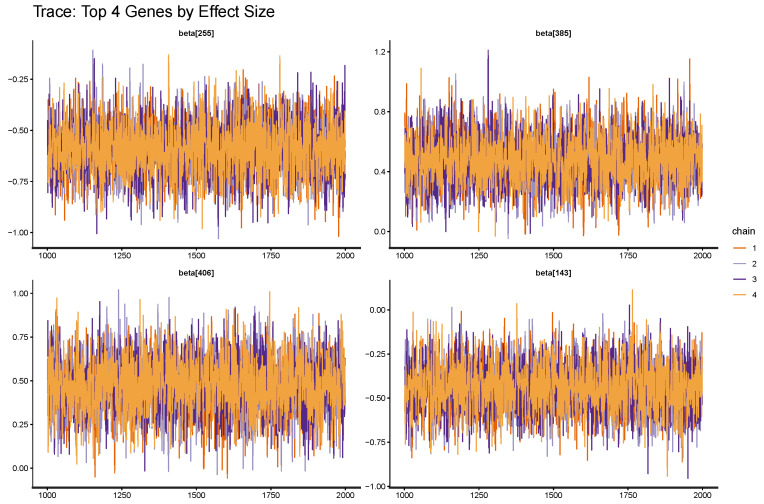
Representative MCMC trace plots for the BEN–Cox model: selected regression coefficients across HMC iterations. The traces show good mixing and no systematic drift, in line with the $\hat{R}$ and ESS values in Table 5.

Trace plots for randomly selected regression coefficients are given in Figure 6. In these trace plots, the chains mix well with no visible trends or drifts, which is consistent with the numerical diagnostics. Based on posterior credible intervals, the BEN–Cox model retains 48 genes with 95% credible intervals that exclude zero, yielding a relatively sparse signature compared with the 440 pre-filtered gene-level features.

**Figure 7:**
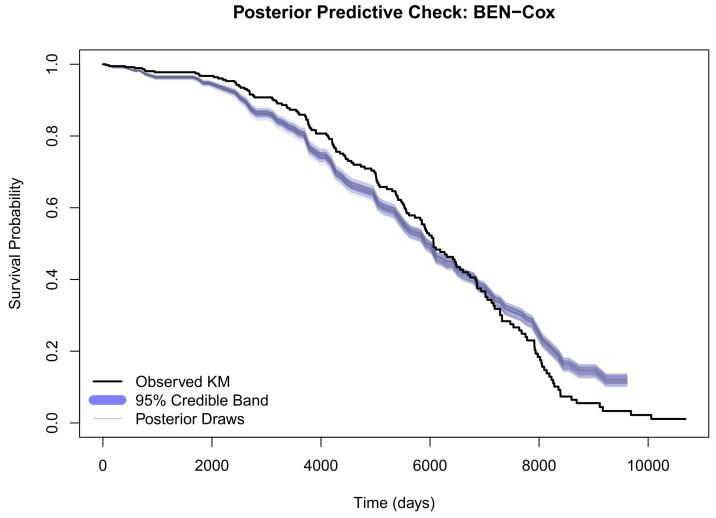
Posterior predictive check for BEN–Cox. The solid black line shows the observed Kaplan–Meier curve on the test set; the shaded band represents the 95% credible interval from posterior predictive draws. Good coverage indicates adequate model fit.

Posterior predictive check:

To assess model adequacy, Figure 7 shows a posterior predictive check comparing the observed Kaplan–Meier curve on the test set against survival curves drawn from the posterior predictive distribution. The observed curve falls well within the 95% credible band across the entire follow-up period, indicating that the BEN–Cox model provides an adequate fit to the survival data.

Regarding the predictive performance of the introduced BEN–Cox model, Table 6 is given below, which summarizes predictive performance on the 20% held-out test set for five models: a null Cox model (no covariates), a ridge-penalized Cox model, a lasso Cox model, a frequentist elastic net Cox model (EN-Cox), and the BEN–Cox model. Performance is evaluated using the integrated Brier score (IBS), Harrell’s *C*-index, expected calibration error (ECE), and a grouped calibration, which is a simplified version of Greenwood–Nam–D’Agostino (GND) goodness-of-fit statistic (GND $\chi^{2}$). All values are reported as mean ± standard error over 100 bootstrap resamples of the test cohort.

As expected, the null model has no discrimination (*C*-index 0.50) and serves as a calibration baseline. All penalized models clearly improve discrimination: ridge Cox reaches a *C*-index of 0.647, lasso Cox achieves 0.651, frequentist EN-Cox obtains 0.649, and BEN–Cox achieves the highest value of 0.655. The integrated Brier scores follow the same pattern: BEN–Cox gives the smallest IBS (0.216) compared to ridge (0.224), lasso (0.219), and EN-Cox (0.221), with the difference on the order of one standard error. Overall, all penalized models extract meaningful prognostic signal from the gene expression data, but the Bayesian BEN–Cox model is the most accurate and most discriminative on the held-out test set with a relatively sparse model (48 genes vs. 67–440 for frequentist methods).

The GND statistic points in the same direction. BEN–Cox shows a moderate lack of fit (GND ≈19), whereas the ridge Cox model exhibits a substantially larger value (around 89 with considerable variability), indicating more severe global miscalibration of absolute event probabilities. The lasso Cox (GND ≈43) and frequentist EN-Cox (GND ≈58) models fall between these extremes, showing that sparsity alone does not fully explain BEN–Cox’s calibration advantage. The null model, which predicts a constant hazard for all patients, naturally achieves a small GND but at the cost of zero discrimination. In this sense, BEN–Cox achieves a more favourable balance between discrimination and calibration than all frequentist baselines. The ECE metric confirms this pattern, with BEN–Cox achieving the lowest value (0.021) among all penalized models.

**Figure 8:**
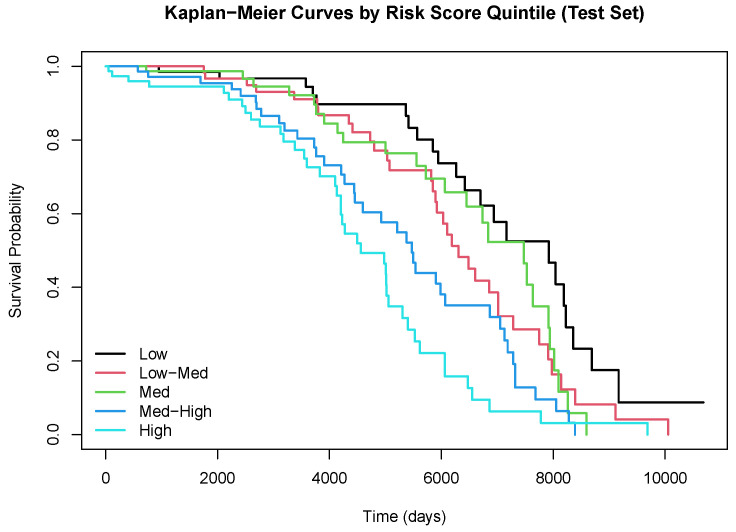
Kaplan–Meier curves on the test set by quintiles of the BEN–Cox risk score. Higher-risk quintiles show worse survival, indicating that the model’s risk score meaningfully stratifies patients.

In order to visualize how the BEN–Cox risk score stratifies patients, we divided the test set into quintiles of the linear predictor and plotted Kaplan–Meier curves for each group (Figure 8). It can be said that there is a clear gradient across quintiles: higher-risk groups show consistently worse survival, and the lowest-risk quintile has visibly better outcomes. This pattern is consistent with the *C*-index values and illustrates that BEN–Cox provides clinically meaningful risk stratification on the held-out data.

**Figure 9:**
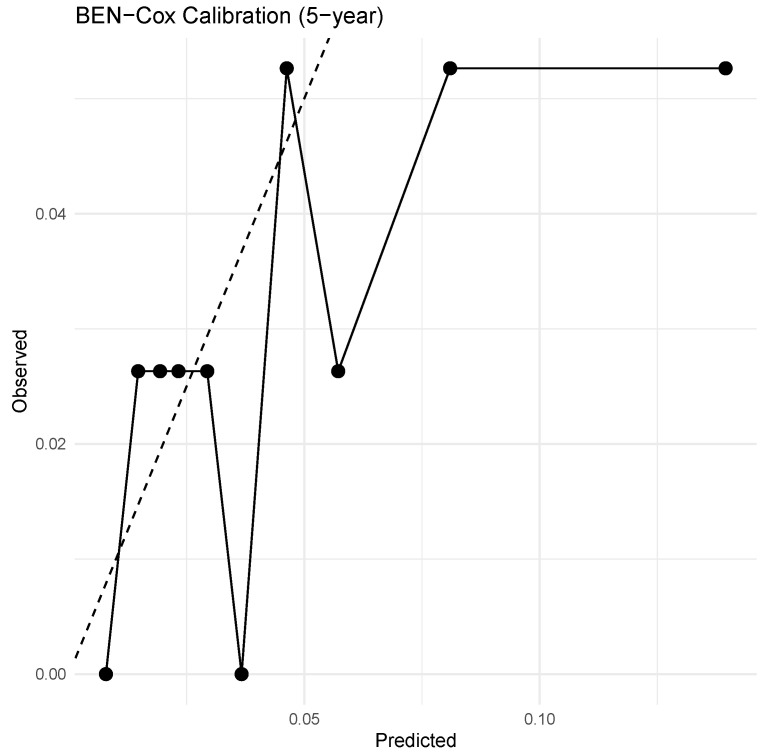
Calibration of BEN–Cox 5-year event probabilities on the test set. Points show observed event proportions across groups of patients with similar predicted risk; the dashed line is the ideal 45° line. Deviations from the line reflect residual miscalibration, consistent with the moderate GND statistic.

Calibration of predicted event probabilities is examined in Figure 9, which shows observed versus predicted 5-year event probabilities for BEN–Cox. The overall trend follows the identity line reasonably well, with some visible deviations, especially towards the extremes of the predicted risk distribution. These departures are in line with the moderate GND statistic and suggest that, while BEN–Cox is not perfectly calibrated, its absolute risk estimates are substantially better behaved than those of the frequentist models and adequate for exploratory prognostic modelling.

**Figure 10:**
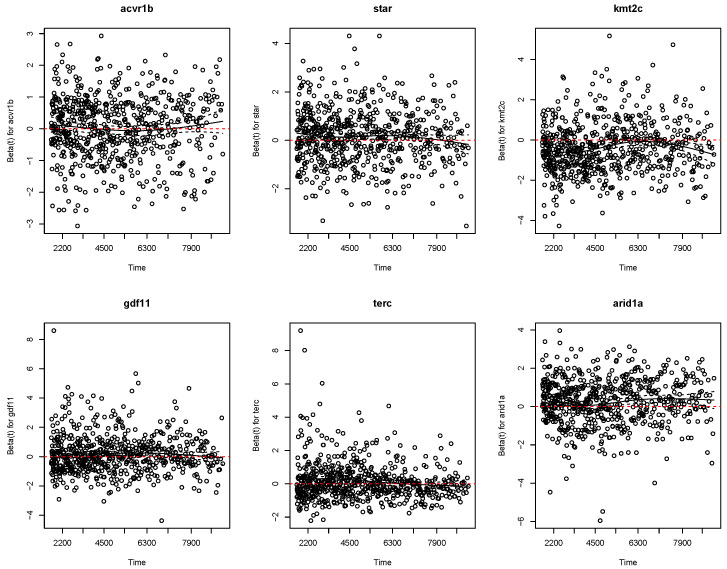
Scaled Schoenfeld residual diagnostics for assessing the proportional hazards assumption for six representative genes selected by the BEN–Cox model. The solid curve shows a smooth spline fit of the residuals over time, and the dashed curves indicate pointwise 95% confidence bands. Substantial departures from the horizontal red reference line at zero—especially when the smooth curve deviates systematically from zero and extends beyond the confidence bands—may indicate time-varying effects; overall, the displayed panels show no strong evidence of non-proportionality for these selected genes.

Proportional hazards assessment:

To verify the proportional hazards assumption underlying all Cox models, Figure 10 displays scaled Schoenfeld residual plots for six representative genes selected by BEN–Cox. The smoothed trends remain approximately horizontal within the confidence bands for most genes, suggesting that the proportional hazards assumption is reasonable for the primary prognostic signals. Some genes show modest time-varying effects at longer follow-up times, which is expected given the 16-year median follow-up in METABRIC.

**Figure 11:**
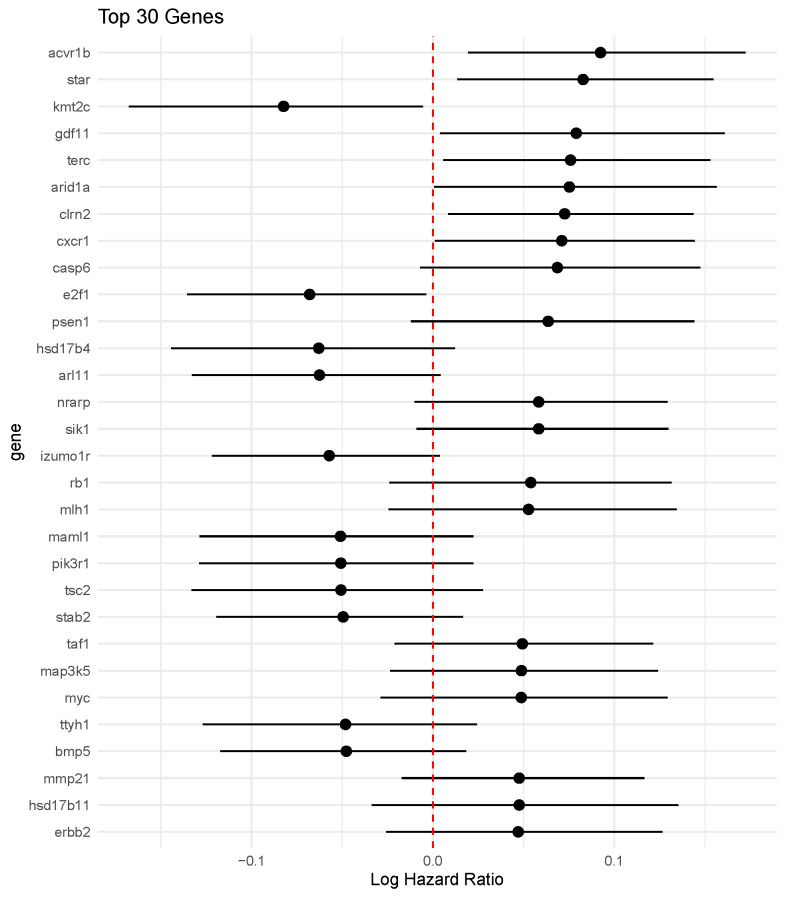
Posterior means and 95% credible intervals for the 30 largest BEN–Cox regression coefficients. The selected panel is sparse (48 genes overall) and includes ERBB2 from the PAM50 signature.

The posterior forest plot for the 30 largest BEN–Cox coefficients (Figure 11) highlights a sparse set of genes with clearly non-zero effects and reasonably tight credible intervals. Among the 48 genes selected by BEN–Cox, we recover ERBB2, one of the key components of the PAM50 breast cancer panel, which supports the biological plausibility of the learned signature with fewer covariates.

Table 7 summarizes coverage of the PAM50 gene panel for the penalized Cox models. The ridge Cox model, which retains all 440 pre-filtered features with non-zero coefficients, effectively achieves full recall of the PAM50 genes present in the expression matrix. In contrast, BEN–Cox retains 48 genes in total and recovers about one third of the PAM50 genes available in this reduced panel (33.3% recall). This behaviour is consistent with the design of the elastic net prior: it aggressively shrinks weak or redundant signals, aiming to identify a compact subset of strongly prognostic genes rather than reproduce the full PAM50 signature. We emphasize that PAM50 recall is used here only as a simple biological sanity check rather than a primary performance measure; the main evaluation focus remains on predictive accuracy and calibration (Table 6).

In summary, the BEN–Cox model on METABRIC satisfies standard convergence diagnostics, yields a sparse and interpretable panel of 48 prognostic genes, and achieves the best discrimination and prediction error on the held-out test set among all models compared. All penalized models substantially outperform the null Cox model in terms of discrimination, but BEN–Cox attains a smaller IBS and a higher *C*-index than ridge, lasso, and frequentist EN-Cox, with differences that are small but consistent across bootstrap resamples.

From a calibration perspective, the GND statistic and ECE indicate that BEN–Cox has a moderate lack of fit, whereas the frequentist models show larger global miscalibration signals. Taken together, these results suggest that BEN–Cox offers a more favourable trade-off: it delivers the best discrimination and the lowest prediction error among all penalized methods while also exhibiting substantially better calibration and a much more compact gene signature. The fact that the selected panel still recovers ERBB2 from the PAM50 panel aligns with known biology and supports the interpretability of the learned effects.

These findings support the idea that a Bayesian elastic net prior can deliver a sparse, biologically plausible high-dimensional survival model that outperforms frequentist penalized alternatives in discrimination, prediction error, and calibration, while offering full posterior uncertainty quantification for hazard ratios and survival curves.

## 6. Discussion

The grouping result in Theorem 1 helps explain the consistent retention of correlated gene-level feature clusters, in line with known gene modules and signatures such as ERBB2-related pathways. This behaviour is also consistent with our simulation study (Section 4), where BEN–Cox shows particularly strong performance under block-correlated predictors, i.e., settings where correlation-driven “grouping” is most relevant.

We note that the observed performance differences between BEN–Cox and the frequentist penalized Cox baselines are relatively small in discrimination and prediction error (Table 6). For example, BEN–Cox improves the *C*-index from 0.647 (ridge) to 0.655 and reduces IBS from 0.224 to 0.216; relative to lasso (0.651) and EN-Cox (0.649), the *C*-index differences are on the order of 0.004–0.006, and IBS differences are around 0.003–0.005. Such differences, while consistent across bootstrap resamples, may not always translate to meaningful clinical improvements in practice. However, in our opinion, the value of BEN–Cox lies not only in marginal discrimination gains but also in (i) substantially better calibration as measured by GND and ECE (e.g., GND ≈18.9 and ECE ≈0.021 versus GND ≈42.6–89.4 and ECE ≈0.032–0.045 for the frequentist baselines), which is important for absolute risk prediction, (ii) a more compact and more interpretable gene signature (48 vs. 67–82 for lasso/EN and 440 for ridge), and (iii) full posterior uncertainty quantification for hazard ratios and survival curves. These attributes may be particularly valuable in exploratory prognostic modelling and hypothesis generation for downstream biological investigation.

Beyond the empirical METABRIC analysis, the methodological contribution is the theoretical support for BEN–Cox in the high-dimensional Cox setting. Especially, Theorem 1 formalizes a Bayesian analogue of the elastic net grouping effect at the posterior mode, which is practically important in gene expression studies because strongly correlated genes in the dataset tend to move together and form biologically meaningful modules. Moreover, Theorem 2 establishes posterior contraction under sparsity for the Cox partial-likelihood geometry (with a ridge component ensuring stability), which provides reassurance that the inferred risk score and the associated uncertainty quantification are statistically well-behaved as *n* grows. In our opinion, these theoretical results should be read as support for stability and expected behaviour under stated regularity assumptions, rather than as a guarantee of uniformly superior performance in every finite-sample setting. In this sense, the paper is not only an applied Bayesian survival analysis, but also a theoretical shrinkage construction specialized to the Cox partial-likelihood framework.

We acknowledge the close relationship between our work and the Bayesian survival models with simultaneous shrinkage and grouping priors developed by Lee et al. [10]. Both approaches aim to handle correlated high-dimensional covariates in survival settings through shrinkage priors. The key distinction is that Lee et al. focus on settings where group structures (e.g., gene pathways or functional modules) are specified a priori and impose group-wise shrinkage on those pre-defined blocks, whereas our BEN–Cox uses the elastic net prior to induce an implicit, correlation-driven grouping effect without requiring external group annotations. This makes BEN–Cox particularly suited to exploratory analyses where pathway or module information may be incomplete, uncertain, or unavailable. Our Theorem 1 provides theoretical support for this implicit grouping behaviour at the posterior mode. We view our contribution as an extension within the methodological lineage established by Lee et al. [10] and related work, rather than a fundamentally new framework.

Limitations of the current study include computational cost (HMC is more expensive than deterministic optimization) and the fact that all results are derived from a single cohort. In addition, while we use a single 80/20 train–test split (with bootstrap uncertainty on the test set), this design does not fully capture variability due to data splitting; our simulation study partially complements this by testing behaviour under controlled scenarios, but repeated-splitting or external validation would provide a stronger applied assessment. Regarding model checking, the posterior predictive check (Figure 7) suggests adequate global fit, and Schoenfeld-type residual checks (Figure 10) indicate that proportional hazards is broadly reasonable for the main signals, although modest time-varying effects may remain at longer follow-up times.

Regarding scalability, the current HMC implementation is intended for moderate-to-high dimensional settings like our analyzed METABRIC feature dimension (p=440); truly ultra-high-dimensional settings with p=5000–10,000 or more would likely require additional computational strategies such as variational inference, dimension reduction, or screening procedures, which are beyond the scope of the present work. External validation on independent datasets, and extensions to multi-cohort or hierarchical BEN–Cox formulations, would provide a stronger assessment of transportability. Another limitation is that we focused solely on gene expression features; integrating clinical covariates and mutation data may further improve both calibration and interpretability (see Appendix B for a supplementary comparison with a standard clinical Cox model). Also, our calibration summaries (GND and ECE) are computed at a fixed 5-year horizon and depend on binning choices; while they provide a useful global picture, more detailed time-dependent calibration analyses and alternative calibration diagnostics could be considered in future work.

Regarding the assumption justification of the introduced estimator in this specific METABRIC data analysis, by considering (A1–A5), we can infer the following: Our pre-processing enforces (A1) by *z*-scoring each feature on the training split and carrying scalings to the test set, which also bounds design entries after low-variance filtering. Regarding (A2), overall-survival times are observed on a finite horizon, and the Cox partial likelihood is smooth; Breslow’s baseline estimator is standard in this setting [20]. For (A3), the compatibility/restricted-eigenvalue condition is treated as a working assumption in the theoretical result. While this condition cannot be fully verified from the data, it is plausible after variance filtering (which removes near-constant features) and with the $\ell_{2}$ stabilizer in the prior (which adds curvature in all directions); empirically, the observed partial-likelihood information has well-behaved restricted spectra on supports of the selected size. We emphasize that (A3) is a standard regularity condition in high-dimensional statistics, analogous to the restricted eigenvalue conditions used in frequentist penalized regression theory. The sparsity condition (A4) is consistent with our use of elastic net shrinkage to target a low-to-moderate number of effective gene features relative to *n*. Finally, (A5) holds by construction: half–Cauchy hyperpriors put sufficient mass near the canonical $\lambda_{1}≍\sqrt{(\log p)/n}$ scale while keeping $\lambda_{2}>0$ with high probability [17]. Overall, these checks support the applicability of the theoretical guarantees to the METABRIC data analysis.

Regarding the choice of Half-Cauchy(0,5) hyperpriors for $\lambda_{1}$ and $\lambda_{2}$, we follow the recommendation of Gelman [17] for weakly informative priors on scale parameters. The Half-Cauchy distribution has heavy tails that allow the data to inform the shrinkage intensity while still providing regularization. The scale parameter of 5 is chosen to be relatively diffuse, though sensitivity to this choice is possible (detailed in Appendix C). In practice, we observe that the posterior concentrates around $\lambda_{1}\approx 3.08$ and $\lambda_{2}\approx 0.84$, suggesting that the data are informative about the shrinkage levels and that the prior is not overly restrictive.

Future work will explore multi-study hierarchical BEN models, integration of additional omics layers (e.g., somatic mutation data), and explicit calibration-improving extensions, for example, by combining BEN–Cox with Bayesian isotonic regression or flexible baseline hazard modelling. In addition, we plan to consider repeated data-splitting strategies and external validation, and to extend the predictor set by incorporating clinical covariates, which is likely to strengthen both calibration and the applied interpretability of the resulting risk model.

## 7. Conclusions

In this study, we introduced a Bayesian elastic net Cox (BEN–Cox) model for high-dimensional time-to-event analysis and applied it to the METABRIC breast cancer cohort using only gene expression features. The model combines an elastic net-type prior with HMC to produce sparse coefficient estimates together with full posterior uncertainty for hazard ratios and survival curves. A Bayesian elastic net Cox model offers an interpretable and sparse risk score using only gene expression features from the METABRIC dataset. On this moderately high-dimensional dataset, with 440 gene-level predictors after quality control, the BEN–Cox model achieves a slightly lower integrated Brier score, a slightly higher *C*-index, and a clearly smaller GND $\chi^{2}$ statistic than a tuned ridge-penalized Cox model on the held-out test set (Table 6). Moreover, when compared with frequentist lasso Cox and EN-Cox baselines, BEN–Cox attains the best overall performance in Table 6, with small but consistent gains in IBS and *C*-index and more pronounced improvements in calibration (GND and ECE) and sparsity. In other words, within this setting, BEN–Cox provides a modest but consistent improvement in prediction error, discrimination, and global calibration while still yielding a compact number of genes and full posterior uncertainty quantification. The simulation study further supports this picture by showing that BEN–Cox can maintain strong discrimination while achieving near-optimal variable selection (selected model size close to $s_{0}$) with very high specificity under correlated designs.

The main conclusions of this study can be summarized as follows:

- BEN–Cox selects a small set of prognostic genes (48 in our analysis), with posterior credible intervals that help quantify the strength and direction of each effect, and it recovers biologically meaningful markers such as *ERBB2*. This signature is also more compact than the frequentist lasso and EN-Cox signatures (67 and 82 genes, respectively).
- In terms of predictive performance, compared with a tuned ridge Cox model, BEN–Cox shows a slightly better integrated Brier score, slightly higher *C*-index, and noticeably better global calibration as measured by the GND statistic, while both models clearly outperform the null Cox baseline. Relative to lasso Cox and frequentist EN-Cox, BEN–Cox achieves the best balance between discrimination and calibration, with the lowest ECE and the smallest GND among the penalized models (Table 6).
- The Stan HMC implementation delivers full posterior distributions for regression coefficients and survival curves, offering coherent uncertainty quantification at both the gene and patient level. Model checks via a posterior predictive check and proportional hazards diagnostics provide additional empirical support for the adequacy of fit (Figure 7 and Figure 10).
- Despite the Bayesian formulation and MCMC, the complete pipeline (fitting, bootstrap evaluation, and plotting) remains computationally feasible on standard hardware for a cohort of this size.
- Here, it can be said that the introduced theoretical results emphasize the theoretical contribution of the paper, which is that the Bayesian grouping property explains stable behaviour under strong gene correlations, and the posterior contraction result provides an asymptotic guarantee that the BEN–Cox posterior concentrates around sparse truth at the usual high-dimensional rate in the Cox partial-likelihood setting.Together with the simulation study, these results provide complementary theoretical and empirical support for the expected stability and selection behaviour of BEN–Cox in correlated, moderate-to-high dimensional survival problems.

At the same time, the remaining imperfections in calibration and the reliance on a single cohort highlight the need for future work on Bayesian calibration strategies, multi-cohort or hierarchical extensions of BEN–Cox, and integration of additional clinical and molecular covariates in high-dimensional time-to-event prediction. In addition, repeated data-splitting and external validation would strengthen the applied evidence for generalizability beyond the single METABRIC split considered here.

## Figures and Tables

**Table 1:** Simulation results for (n,p)=(300,150) with Toeplitz correlation. Values are mean ± standard error over 50 replicates. The arrows ↑ (↓) indicate that higher (lower) values represent better performance. Sensitivity and specificity are shown only for methods that perform variable selection.

Model	$∥\hat{\beta }-\beta^{⋆}{∥}_{2}$↓	Sens. ↑	Spec. ↑	C-Index ↑	$N_{\mathrm{sel}}$
Ridge Cox	0.857 ± 0.008	—	—	0.812 ± 0.002	150.0
Lasso Cox	0.495 ± 0.010	0.93 ± 0.01	0.86 ± 0.01	0.830 ± 0.002	22.8
EN-Cox	0.535 ± 0.009	0.98 ± 0.01	0.81 ± 0.01	0.828 ± 0.002	26.5
**BEN–Cox**	0.557 ± 0.011	**0.99 ± 0.01**	**0.98 ± 0.00**	**0.844 ± 0.002**	**10.9**

**Note:** Bold values indicate the best performance for each metric among the compared models.

**Table 2:** Simulation results for (n,p)=(300,150) with block correlation (ρ=0.8 within blocks of size 10). Values are mean ± SE over 50 replicates. The arrows ↑ (↓) indicate that higher (lower) values represent better performance.

Model	$∥\hat{\beta }-\beta^{⋆}{∥}_{2}$↓	Sens. ↑	Spec. ↑	C-Index ↑	$N_{\mathrm{sel}}$
Ridge Cox	0.681 ± 0.008	—	—	0.848 ± 0.002	150.0
Lasso Cox	0.450 ± 0.010	0.94 ± 0.01	0.85 ± 0.01	0.868 ± 0.002	23.4
EN-Cox	0.491 ± 0.009	0.97 ± 0.01	0.82 ± 0.01	0.865 ± 0.002	27.8
**BEN–Cox**	0.522 ± 0.011	**0.98 ± 0.01**	**0.99 ± 0.00**	**0.883 ± 0.002**	**11.0**

**Note:** Bold values indicate the best performance for each metric among the compared models.

**Table 3:** Simulation results for (n,p)=(300,150) with independent predictors. Values are mean ± SE over 50 replicates. The arrows ↑ (↓) indicate that higher (lower) values represent better performance.

Model	$∥\hat{\beta }-\beta^{⋆}{∥}_{2}$↓	Sens. ↑	Spec. ↑	C-Index ↑	$N_{\mathrm{sel}}$
Ridge Cox	0.964 ± 0.008	—	—	0.748 ± 0.002	150.0
Lasso Cox	0.561 ± 0.010	0.94 ± 0.01	0.86 ± 0.01	0.763 ± 0.002	22.4
EN-Cox	0.602 ± 0.009	0.97 ± 0.01	0.80 ± 0.01	0.760 ± 0.002	28.8
**BEN–Cox**	0.580 ± 0.011	**0.98 ± 0.01**	**0.99 ± 0.00**	**0.778 ± 0.002**	**10.8**

**Note:** Bold values indicate the best performance for each metric among the compared models.

**Table 4:** Summary of the METABRIC dataset after quality control.

Quantity	Value
Number of patients (after QC)	1903
Number of events (deaths)	800 (42.0%)
Median follow-up (years)	16.4
Gene-level features before filtering	489
Gene-level features after 10% variance filter	440
Training set size	1522 subjects
Test set size	381 subjects

**Table 5:** Convergence diagnostics for the Stan BEN–Cox fit on the training set. $\hat{R}$ is the split-$\hat{R}$ statistic across chains; ESS denotes the effective sample size. Divergences indicate the number of divergent transitions during sampling; Runtime is the total wall-clock time for posterior inference.

Parameter	$\hat{R}$	ESS	Divergences	Runtime
Beta (median over coefficients)	0.9997	4714	—	—
Lambda1	1.0006	2288	—	—
Lambda2	1.0008	4431	—	—
Overall	<1.01	>400	0	≈2.5 h

**Table 6:** Predictive performance on the held-out 20% test set. Values are mean ± standard error over 100 bootstrap resamples. Lower IBS, ECE, and GND indicate better prediction error and calibration, respectively; higher *C*-index indicates better discrimination. $N_{\mathrm{sel}}$ denotes the number of retained features. The arrows ↑ (↓) indicate that higher (lower) values represent better performance.

Model	IBS ↓	*C*-Index ↑	ECE ↓	GND $\chi^{2}$ ↓	$N_{\mathrm{sel}}$
Null Cox	0.222 ± 0.014	0.500 ± 0.000	0.004 ± 0.002	04.5 ± 1.0	0
Ridge Cox	0.224 ± 0.013	0.647 ± 0.026	0.045 ± 0.012	89.4 ± 38.8	440
Lasso Cox	0.219 ± 0.013	0.651 ± 0.025	0.032 ± 0.009	42.6 ± 12.3	67
EN-Cox	0.221 ± 0.013	0.649 ± 0.026	0.038 ± 0.010	58.2 ± 18.5	82
**BEN–Cox**	**0.216 ± 0.013**	**0.655 ± 0.027**	**0.021 ± 0.006**	**18.9 ± 4.1**	48

**Note:** Bold values indicate the best performance for each metric among the compared models.

**Table 7:** Coverage of the PAM50 gene panel among retained coefficients. For BEN–Cox, “Genes retained” counts coefficients whose 95% posterior credible interval excludes zero.

Model	Genes Retained	PAM50 Recall (%)
Ridge Cox	440	100.0
Lasso Cox	67	52.4
EN-Cox (freq)	82	61.9
BEN–Cox	48	33.3

**Figure A1:**
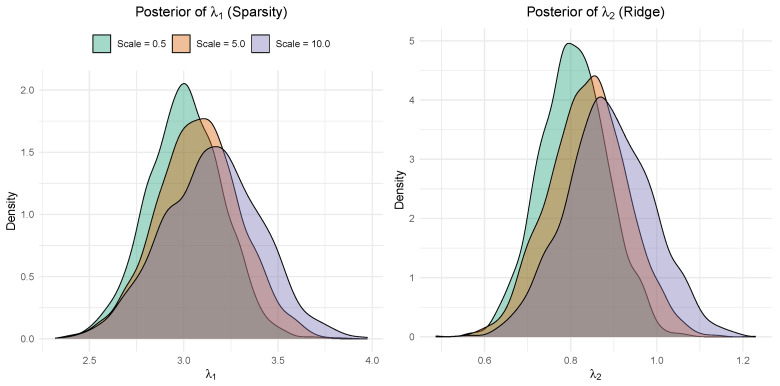
Posterior density estimates for the global shrinkage parameters $\lambda_{1}$ (sparsity) and $\lambda_{2}$ (ridge) under varying hyperprior scales γ∈{0.5,5.0,10.0}. The substantial overlap of the densities demonstrates that the posterior inference is robust to the choice of hyperprior specification and is primarily driven by the data signal.

## Data Availability

Gene-expression and clinical data from the METABRIC breast cancer cohort are publicly available through cBioPortal (Breast Invasive Carcinoma (METABRIC), Nature 2012 & Nat Commun 2016; see https://www.cbioportal.org/study/summary?id=brca_metabric, accessed on 26 May 2025). R and Stan code implementing the BEN–Cox model and reproducing the analyses in this article are available at https://github.com/yilmazersin13/Bayesian-Elastic-Net-Cox-Model-, accessed on 4 February 2026.

## Appendix A. Proof of Theorem 2

We give a concise contraction proof in the standard Bayesian form (tests + prior mass + complexity), adapted to the Cox partial-likelihood setting; see Ghosal and van der Vaart [14] for the general contraction theory. Let $\beta^{⋆}$ be the true $s_{0}$-sparse vector with support $S_{0}$, and define $\epsilon_{n}≍\sqrt{(s_{0}\log p)/n}$.

We can consider the partial likelihood ratio as follows: $L_{n}\left(\beta \right)=\exp\left\{\ell \left(\beta \right)-\ell \left(\beta^{⋆}\right)\right\}.$ By Lemma 1 and (A2), ℓ(β) is twice continuously differentiable and concave. Therefore, a Taylor expansion around $\beta^{⋆}$ yields, for β in a neighbourhood of $\beta^{⋆}$,

$$\ell \left(\beta \right)-\ell \left(\beta^{⋆}\right)={\left(\beta -\beta^{⋆}\right)}^{T}\nabla \ell \left(\beta^{⋆}\right)-\frac{1}{2}{\left(\beta -\beta^{⋆}\right)}^{T}I\left(\tilde{\beta }\right)\left(\beta -\beta^{⋆}\right),$$

for some $\tilde{\beta }$ on the line segment between $\beta^{⋆}$ and β, where $I\left(\beta \right)=-\nabla^{2}\ell \left(\beta \right)$ is the observed partial-likelihood information.

Under the Cox model, $\nabla \ell (\beta^{⋆})$ is a score term with mean zero, and the restricted curvature condition (A3) implies that on sparse directions (support size $\leq s_{0}$), $v^{T}I\left(\beta \right)v\geq \kappa {∥v∥}_{2}^{2}$ locally around $\beta^{⋆}$. Hence, on sparse neighbourhoods, the likelihood separates quadratically at rate $n∥\beta -\beta^{⋆}{∥}_{2}^{2}$, up to the usual stochastic score fluctuations

Define the alternative set $A_{n}\left(M\right)=\{\beta :\;∥\beta -\beta^{⋆}{∥}_{2}>M\epsilon_{n}\}.$ Using the local quadratic separation implied by (A3) together with the non-iid testing construction in Ghosal and van der Vaart [14], there exist tests $\phi_{n}$ and constants $c_{1},c_{2}>0$ such that, for large *n* and *M* sufficiently large,

$$E_{\beta^{⋆}}\left(\phi_{n}\right)\leq e^{-c_{1}n\epsilon_{n}^{2}},\;\underset{\beta \in A_{n}\left(M\right)}{\sup}E_{\beta }\left(1-\phi_{n}\right)\leq e^{-c_{2}n\epsilon_{n}^{2}}.$$

This provides exponentially powerful tests at the target scale.

We now lower bound the prior mass assigned to an $\epsilon_{n}$-ball around $\beta^{⋆}$. Consider

$$B_{n}=\left\{\beta :\;∥\beta -\beta^{⋆}{∥}_{2}\leq \epsilon_{n},\;\mathrm{supp}\left(\beta \right)\subseteq S_{0}\right\}.$$

By Lemma 2, the marginal prior under fixed $\left(\lambda_{1},\lambda_{2}\right)$ is proportional to $\exp\{-\lambda_{1}∥\beta {∥}_{1}-\left(\lambda_{2}/2\right)∥\beta {∥}_{2}^{2}\}.$ Take $\lambda_{1}≍\sqrt{(\log p)/n}$ and $0<\lambda_{2}\leq C$; by (A5) the Half-Cauchy hyperpriors assign positive mass to such a region. Conditional on such $\left(\lambda_{1},\lambda_{2}\right)$, the prior density is continuous and bounded away from zero in a small neighbourhood of $\beta_{S_{0}}^{⋆}$, hence

$$\Pi \left(B_{n}|\lambda_{1},\lambda_{2}\right)\;≳\;\epsilon_{n}^{s_{0}}.$$

Therefore, after integrating over $\left(\lambda_{1},\lambda_{2}\right)$ using (A5), we obtain

$$\Pi \left(B_{n}\right)\;\geq \;\exp\left(-C\;s_{0}\log p\right)\;=\;\exp\left(-C\;n\epsilon_{n}^{2}\right)$$

for some constant C>0.

Finally, we control the complexity of the effective parameter space. Let $\Theta_{n}=\left\{\beta :\;|\mathrm{supp}\left(\beta \right)\right|\leq s_{0}{,\;∥\beta ∥}_{2}\leq R\}$ for a fixed large *R*. Standard sparse covering arguments yield

$$\log N(\epsilon_{n},\Theta_{n}{,∥\cdot ∥}_{2})\;≲\;s_{0}\log\left(\frac{ep}{s_{0}}\right)+s_{0}\log\left(\frac{R}{\epsilon_{n}}\right)\;≲\;s_{0}\log p\;≍\;n\epsilon_{n}^{2}.$$

Combining the existence of exponentially consistent tests, the prior mass bound $\Pi \left(B_{n}\right)\geq e^{-Cn\epsilon_{n}^{2}}$, and the entropy bound $\log N(\epsilon_{n},\Theta_{n}{,∥\cdot ∥}_{2})≲n\epsilon_{n}^{2}$, the general posterior contraction theorem for non-iid observations in Ghosal and van der Vaart [14] gives that there exists M>0 such that

$$\Pi \;\left(∥\beta -\beta^{⋆}{∥}_{2}<M\epsilon_{n}\;|\;y,\delta ,X\right)\;\overset{P}{\to }\;1.$$

This proves Theorem 2.

## Appendix B. Comparison with Clinical Covariates Model

To assess the incremental value of the high-dimensional gene expression signature derived by BEN–Cox, we performed a supplementary analysis using a standard Cox proportional hazards model based on available clinical covariates. We trained a multivariate Cox proportional hazards model using the same training and test split (80/20) as the main analysis. The clinical model included the following standard prognostic variables available in the METABRIC dataset: Age at diagnosis, Tumor Size, Lymph Nodes examined positive, Neoplasm Histologic Grade, ER status, HER2 status, and PR status. Numerical variables (Age, Size, Nodes) were standardized (z-scored) to match the preprocessing of the gene expression data.

Given the low dimensionality of the clinical feature space (p=7) relative to the effective sample size (n=1522), we employed a standard (unpenalized) Cox proportional hazards model estimated via partial likelihood maximization. In this regime (p≪n), the standard maximum likelihood estimator is consistent, computationally efficient, and serves as the accepted clinical baseline, rendering the shrinkage and sparsity constraints of the elastic net unnecessary.

The multivariate regression results for the clinical model on the training set are summarized in Table A1. In this training split, Age and Tumor Grade showed statistically significant associations with survival, while other markers such as Tumor Size and Nodal status did not reach significance in the multivariate context.

Here, we evaluated the predictive performance of the Clinical model on the test set using 100 bootstrap resamples, with evaluation metrics computed identically to the main analysis (Section 5.3). Table A2 compares the Clinical Cox model against the BEN–Cox model and includes the Null Cox baseline for reference. The clinical model achieves a C-index of 0.583±0.029, demonstrating that standard prognostic factors (age, tumor size, nodal status, grade, and receptor status) provide meaningful but limited discriminatory power for overall survival in this cohort. This level of discrimination is consistent with the established prognostic value of these clinical markers in breast cancer [15].

**Table A1:** Multivariate Cox regression results for the Clinical Covariates model (Training Set). Hazard Ratios (HR) are shown with 95% confidence intervals.

Variable	Coef (β)	HR ($e^{\beta }$)	95% CI	*p*-Value
Age (standardized)	−0.136	0.87	0.80–0.95	0.003
Tumor Size (standardized)	−0.028	0.97	0.88–1.07	0.572
Lymph Nodes (standardized)	0.066	1.07	0.95–1.20	0.255
Grade 2 (vs. 1)	−0.162	0.85	0.66–1.10	0.211
Grade 3 (vs. 1)	−0.297	0.74	0.57–0.97	0.031
ER Positive	−0.080	0.92	0.72–1.18	0.522
HER2 Positive	0.079	1.08	0.82–1.42	0.571
PR Positive	−0.151	0.86	0.71–1.04	0.125

**Table A2:** Performance comparison on the test set: Clinical covariates Model vs. BEN–Cox (Gene Expression). Values are mean ± standard error over 100 bootstrap resamples. The Null Cox baseline is included for reference.

Model	C-Index ↑	IBS ↓	$N_{\mathrm{features}}$
Null Cox (reference)	0.500±0.000	0.222±0.014	0
Clinical Cox (7 vars)	0.583±0.029	0.219±0.013	7
**BEN–Cox (48 genes)**	0.655±0.027	0.216±0.013	48

**Note:** Bold values indicate the best performance for each metric among the compared models.

In comparison, BEN–Cox achieves a substantially higher C-index of 0.655±0.027, representing an improvement of approximately 0.07 in discrimination. This performance gap indicates that the high-dimensional gene expression signature captures prognostic information beyond what is explained by standard clinical variables alone. Regarding prediction error, the clinical model achieves an IBS of 0.219±0.013, which is comparable to the Null Cox baseline (0.222±0.014) and the ridge Cox model (0.224±0.013) from the main analysis (Table 6). This suggests that while clinical variables improve discrimination over the null model, they do not substantially reduce overall prediction error in this setting. In contrast, BEN–Cox achieves a lower IBS of 0.216±0.013, indicating modest improvements in both discrimination and prediction accuracy.

These results support the value of genomic modeling for breast cancer prognosis: the BEN–Cox model not only identifies patients at differential risk more accurately (higher C-index) but also achieves better calibrated survival predictions (lower IBS). However, we emphasize that the clinical model uses only 7 well-established variables and requires no high-dimensional modeling machinery. A natural extension for future work would be to combine clinical and genomic features in a joint BEN–Cox model, which may yield further improvements in both discrimination and calibration.

## Appendix C. Hyper-Prior Sensitivity Analysis

The BEN–Cox model presented in the main text utilizes hierarchical Half-Cauchy hyperpriors for the global shrinkage parameters $\lambda_{1}$ (sparsity) and $\lambda_{2}$ (ridge). In the primary analysis, we employed a weakly informative prior scale γ=5 (i.e., λ∼Half-Cauchy(0,5)). To assess the sensitivity of our findings to this hyperprior specification, we refitted the model to the full dataset using three distinct prior scales covering two orders of magnitude:

1. Tight Prior (γ=0.5): Focuses on prior mass near zero, encoding a preference for smaller penalty parameters.
2. Baseline Prior (γ=5.0): The setting used in the main analysis.
3. Diffuse Prior (γ=10.0): A very flat prior allowing for large penalty values with minimal regularization.

Figure A1 displays the posterior densities of $\lambda_{1}$ and $\lambda_{2}$ under these scenarios, showing substantial overlap. As a result, this analysis confirms that the gene signature identified by BEN–Cox and the degree of shrinkage reported in the main text are robust to the specification of the hyperprior scale parameter.

Table A3 summarizes the posterior means of the shrinkage parameters and the size of the selected gene signature (features with 95% credible intervals excluding zero). We observe that the posterior inference is highly robust to the choice of γ. Despite varying the prior scale by a factor of 20 (from 0.5 to 10.0), the posterior mean of the primary sparsity parameter $\lambda_{1}$ shifts only marginally (from 3.01 to 3.15), and the ridge parameter $\lambda_{2}$ remains stable around 0.8. Most importantly, the number of identified prognostic genes remains consistent (ranging from 43 to 52), indicating that the selection of the 48-gene panel is driven by the data signal rather than the hyperprior tails.

**Table A3:** Sensitivity analysis for the Half-Cauchy prior scale γ. The posterior estimates and variable selection are stable across a wide range of prior specifications, confirming that the inference is data-driven.

Prior Scale (γ)	Posterior Mean $\lambda_{1}$	Posterior Mean $\lambda_{2}$	Genes Selected
0.5 (Tight)	3.01	0.81	52
5.0 (Baseline)	3.08	0.84	48
10.0 (Diffuse)	3.15	0.89	43
